# The nodal positivity rate in breast pCR patients with initially, clinically node-negative breast cancer after neoadjuvant systemic therapy: A systematic review and meta-analysis

**DOI:** 10.3389/fonc.2023.1167912

**Published:** 2023-03-29

**Authors:** Le Ma, Heyan Chen, Jianjun He, Peiling Xie, Pin Gao, Yijun Li, Huimin Zhang, Zhimin Fan

**Affiliations:** ^1^ Department of Breast Surgery, General Surgery Center, the First Hospital of Jilin University, Changchun, Jilin, China; ^2^ Department of Breast Surgery, The First Affiliated Hospital of Xi’an Jiaotong University, Xi’an, Shaanxi, China

**Keywords:** breast cancer, neoadjuvant systemic therapy, axillary lymph node positive rate, sentinel lymph node biopsy, meta- analysis

## Abstract

**Background:**

The axillary lymph node positive (ypN+) rate in patients with clinically node-negative (cN0) breast cancer who have achieved breast pathologic complete response (bpCR) after neoadjuvant systemic therapy (NST) is extremely low, and this population has the potential to be exempt from sentinel lymph node biopsy (SLNB). However, an overview of the ypN+ rate in this population for different breast cancer subtypes is lacking.

**Objective:**

To provide the pooled ypN+ rate in cN0 patients who achieved bpCR after NST in different breast cancer subtypes defined by hormone receptor (HR) status and human epidermal growth factor receptor 2 (HER2) status.

**Methods:**

A systematic literature search was conducted in Embase and PubMed on July 20, 2022. Two authors independently selected studies that met the inclusion criteria and extracted all data. The pooled ypN+ rates for each subtype were calculated by a random-effects model using the Stata 16.0 *metaprop* command.

**Results:**

The pooled analysis of 9609 cN0 patients who achieved bpCR showed that the ypN+ rate was lowest for the HR+/HER2+ (0%) subtype, followed by HR+/HER2- (5.1%), HR-/HER2+ (0.6%), and HR-/HER2- (0.3%). Additionally, 6571 cT_1_-T_2_N0 patients who achieved bpCR had a pooled ypN+ rate of 0.6%, and the ypN+ rates for different subtypes were as follows: HR+/HER2+ (1.7%), HR+/HER2- (2.7%), HR-/HER2+ (0.1%), and HR-/HER2- (0.8%).

**Conclusion:**

Our results suggested that cN0 patients who achieve bpCR may be exempt from axillary surgery in the HR+/HER2-, HR+/HER2+, and HR-/HER2- subtypes because of the extremely low probability of residual axillary lymph node disease. However, the safety of omitting axillary surgery needs to be further confirmed by prospective studies.

**Systematic Review Registration:**

https://www.crd.york.ac.uk/PROSPERO/#recordDetails, identifier CRD42022351739.

## Introduction

To provide successful disease control and to enhance the long-term quality of life of patients, the current trend in axillary management for those with early breast cancer is to focus on accuracy and safety. With the addition of targeted therapy to neoadjuvant systemic therapy (NST), the pathological complete response rate (pCR) in breast cancer patients has greatly improved, providing an opportunity to reduce or possibly eliminate surgery for certain patients (1, 2).

At present, sentinel lymph node biopsy (SLNB) is the standard of care and has replaced axillary lymph nodes dissection (ALND) as a staging procedure in clinically node-negative (cN0) patients. SLNB alone without further ALND has been found to be an appropriate, safe, and effective treatment for patients with clinically node-negative (cN0) breast cancer, as demonstrated by the fact that overall survival, disease-free survival, and regional control are not significantly different between the SLNB plus ALND group and the SLNB group (3). However, SLNB after NST remains controversial. Partial evidence has shown that SLNB is feasible in cN0 patients after NST, while the false-negative rate (FNR) can be as high as 15% in cN1 patients after NST (4). How to reduce the FNR and improve the accuracy of SLNB in these patients still needs to be further explored.

This study focused on patients with cN0 after NST. For cN0 patients, the nodal positivity (ypN+) rate following NST is low, particularly in those with breast pCR (bpCR) (5–9). It has been shown that ypN+ rates are less than 2% in patients with TNBC or HER2+ disease and bpCR (7, 10). Barron et al. (10) (n = 5377) and Tadros et al. (7) (n = 116) studied the ypN+ rate in cN0 patients with bpCR after NST and demonstrated that the ypN+ rate was 1.6% for both HER2+ and TNBC. Samiei et al. (9) (n = 986) reported that the ypN+ rate in cN0 patients with bpCR was 6.7%, 1.6%, 0%, and 1.5% in ER+/HER2-, ER+/HER2+, ER-/HER2+ and TNBC, respectively. The Moreover, the GANEA2 study showed that cN0 patients were followed up with SLNB for 3 years after neoadjuvant chemotherapy, with only one recurrence (11). Therefore, it is safe for cN0 patients to achieve bpCR for SLNB after NST. At this time, we raised a question: if the ypN+ ratio of the HER2+ and TNBC subtypes of breast cancer is less than 4%, can SLNB be exempted directly?

Therefore, the aim of this study was to pool and systematically review the ypN+ rate in cN0 patients with bpCR after NST in different subtypes, thereby providing clinicians with medical-based evidence on the safety and potential feasibility of SLNB exemption for such patients.

## Methods

### Literature search strategy

On July 20, 2022, studies evaluating the axillary pathological complete response (apCR) and/or nodal positivity rate (ypN+) for various breast cancer subtypes in patients with cN0 were originally searched in Embase and PubMed. Details of both search strategies are provided in the **Supplementary Materials**. This review protocol (No. CRD42014012901), which was registered in the International Prospective Register of Systematic Reviews (PROSPERO), adhered to the Preferred Reporting Items for Meta-Analyses (PRISMA).

### Eligibility criteria for study inclusion

The following requirements had to be met for studies to be included in this review: patients who were clinically node-negative at the time of diagnosis underwent neoadjuvant chemotherapy, with or without HER2-targeted therapy, with achieved bpCR, followed by SLNB, ALND, or SLNB+ALND. The absence of suspicious or unusual lymph nodes on physical examination or ultrasound imaging was referred to as clinically node-negative. The definition of bpCR was no invasive disease (ypT0 or ypTis) by final pathologic result, and apCR was defined as ypN0/itc or ypN0 by final pathologic result. Second, among individuals with cN0, the ypN0/itc or axillary nodal positivity (ypN+) rates were reported for two or more distinct subtypes of breast cancer. Third, studies using SLNB performed prior to NST or neoadjuvant endocrine or radiation therapy were excluded. Additionally, we considered only English-language cohort studies, case−control studies, and randomized clinical trials.

### Outcome measures

The rate of ypN+ following NST for various breast cancer subtypes was the study’s primary outcome. Micrometastatic or macrometastatic nodal disease was defined as ypN+. It should be emphasized that according to the attribution of isolated tumor cells (itc), the definitions of apCR in the included literature are different. Some studies define apCR as ypN0, while others define it as ypN0/itc. Therefore, if apCR was reported as an outcome event in the included literature and the ypN+ rate was not directly reported, we could also use the formula ypN+ (%) = 100%-ypN0 (or ypN0/itc) (%). To date, the definition of apCR as ypN0 or ypN0/itc is controversial among different research institutions. Conflicting results have been reported regarding the prognostic implications of ypN0 and residual isolated tumor cells.

### Study selection

Two authors independently evaluated all available studies and resolved disagreements by reaching consensus (Le Ma and Heyan Chen). The Newcastle−Ottawa Scale (NOS) was utilized to appraise the validity of eligible studies (12). When two reviewers were uncertain about the quality assessment of a review, they emailed or interviewed the authors to resolve the quality differences.

### Data extraction and analysis

The two reviewers separately retrieved the following study features from the included studies. Characteristics of studies, such as year, first author, research type, and country, were collected. Traits of participants, including cT category, cancer subtype, neoadjuvant systemic therapy regimens, axillary surgery, definition of bpCR and apCR, were extracted. Data extraction disagreements were settled *via* a consensus meeting.

The overall pooled estimate of the ypN+ rate for each subtype was computed using the Stata 16.0 *metaprop* command and the random-effects model for meta-analysis. Subgroup analyses were performed according to different apCR definitions. Forest plots were used to display the estimated variance in size estimates of the ypN+ effect with 95% CI and weights for each subtype. The statistical heterogeneity was measured using the I^2^ statistic, and values of 25%, 50%, and 75% were considered to be low, moderate, and high, respectively. The statistical heterogeneity was evaluated using the χ^2^ test. A two-sided P < 0.05 was regarded as statistically significant.

## Results

### Selected studies and methodological quality

**Figure 1:**
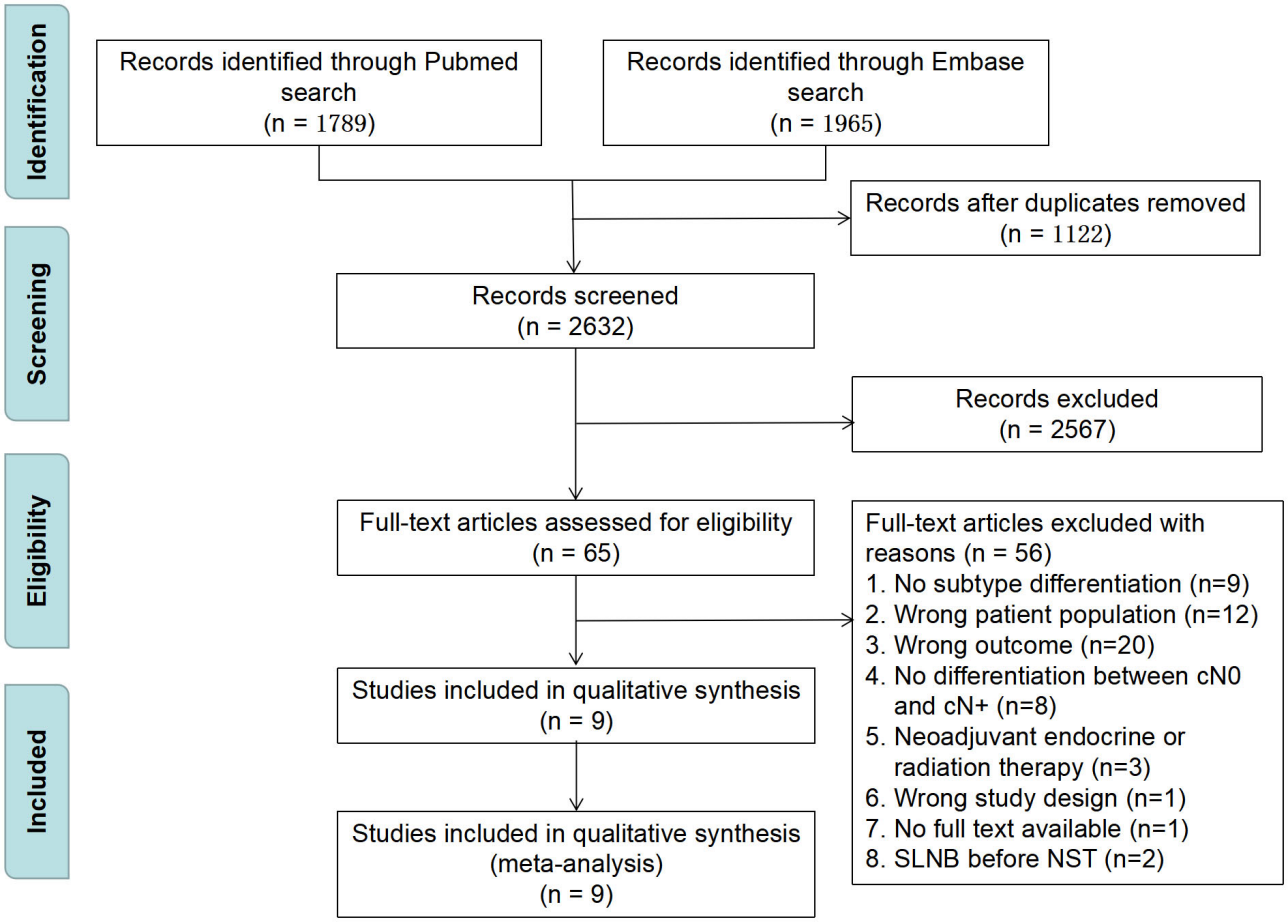
PRISMA flow diagram for study selection. SLNB, sentinel lymph node biopsy; NST, neoadjuvant systematic therapy.

**Figure 1** shows a flow diagram of the literature review procedure. The PubMed and Embase databases yielded a total of 3754 items, and 1122 duplicate papers were eliminated after being loaded into EndNote. There were 65 articles left after the initial screening of titles and abstracts using inclusion and exclusion criteria. By reading the entire articles, 56 items were ultimately eliminated, while 9 were enrolled. The quality of the included studies was evaluated using the NOS, and the outcomes are given in **Supplementary Materials Table S1**.

### Characteristics of studies and participants

A total of 21521 participants were enrolled in the meta-analysis across 9 studies (7, 9, 10, 13–18) (**Table 1**), including 9609 cN0 patients who achieved bpCR. The definition of apCR was ypN0 in 4 studies (7, 14, 15, 17) and ypN0/itc in 5 studies (9, 10, 13, 16, 18). Three of these studies were carried out in the United States (7, 10, 13), three in China (14, 16, 17), one in the Netherlands (9), one in Korea (18), and one in Spain (15). Six retrospective studies and three prospective studies were included. Four studies (7, 9, 10, 15) reported the ypN+ rate under different T stages, among which T1-T2 accounted for 99% (6571/6632). ER+/PR+, ER+/PR- or ER-/PR+ is defined as HR+, and ER-/PR- is defined as HR-.

**Table 1:** General characteristics of the studies included in the meta-analysis.

Source	Country	Centre	Study type	No. of participants with N0	pN0/cN0	cT	Cancer subtype	NST	Axillary surgery	Definition of breast pCR	Definition of apCR
Weiss et al. (13)	US	Single	Prospective	241	cN0	1-4	HER2+ TNBC	1.lapatinib, trastuzumab, or lapatinib + trastuzumab; with paclitaxel 2. carboplatin and/or bevacizumab to paclitaxel, followed by dose-dense doxorubicin and cyclophosphamide ± bevacizumab	SLNB, ALND	ypT0/ypTis	ypN0/itc.
Hong et al. (14)	China	Single	Retrospective	457	pN0/cN0	1-2	^a^ Luminal-A ^b^ Luminal-B (HER2–) ^c^ Luminal-B (HER2+) HER2+ TNBC	Anthracycline and/or taxane with or without trastuzumab ± pertuzumab	SLNB, ALND	ypT0/ypTis	ypN0
Esgueva et al. (15)	Spain	Multiple	Prospective	265	pN0/cN0	1-4	^a^ Luminal-A ^b^ Luminal-B (HER2–) ^c^ Luminal-B (HER2+) HER2+ TNBC	Pertuzumab and trastuzumab ± anthracycline; Lapatinib with trastuzumab	SLNB, ALND SLNB + ALND	ypT0/ypTis	ypN0
Choi et al. (18)	Korea	Single	Retrospective	200	pN0/cN0	1-3	HR+/HER2− HR+/HER2+ HR-/HER2+ TNBC	anthracycline and/or taxane + cyclophosphamide or trastuzumab	SLNB, ALND	ypT0/ypTis	ypN0/itc.
Zhu et al. (17)	China	Multiple	Retrospective	406	pN0/cN0	1-4	HR+/HER2− HR+/HER2+ HR-/HER2+ TNBC	NR	NR	ypT0/ypTis	ypN0
Barron et al. (10)	US	Single	Retrospective	18 093	pN0	1-2	HR+/HER2− HR+/HER2+ HR-/HER2+ TNBC	NR	NR	ypT0/ypTis	ypN0/itc.
Chen et al. (16)	China	Single	Retrospective	53	pN0/cN0	1-4	HR+/HER2− HR+/HER2+ HR-/HER2+ TNBC	Anthracycline and/or taxane with or without trastuzumab	SLNB, ALND	ypT0/ypTis	ypN0/itc.
Samiei et al. (9)	Netherlands	Single	Retrospective	1674	pN0/cN0	1-3	^d^ ER+/HER2- ^e^ ER+/HER2+ ^f^ ER-/HER2+ TNBC	Taxotere, Adriamycin, Cyclophosphamide; Fluorouracil (5FU), Epirubicin, Cyclophosphamide;Adriamycine, Cyclophosphamide with paclitaxel or docetaxel; with or without trastuzumab	SLNB, ALND	ypT0/ypTis	ypN0/itc.
Tadros et al. (7)	US	Single	Prospective	132	pN0/cN0	1-2	HER2+ TNBC	Anthracycline and/or taxane with or without trastuzumab ± pertuzumab	NR	ypT0/ypTis	ypN0

^a^Luminal A was classified as HR+/HER2-; ^b^Luminal B (HER2-) was classified as HR+/HER2-; ^c^Luminal B (HER2+) was classified as HR+/HER2+; ^d^ER+/HER2- was classified as HR+/HER2-; ^e^ER+/HER2+ was classified as HR+/HER2+; ^f^ER-/HER2+ was classified as HR-/HER2.

### HR+/HER2- breast cancer

**Figure 2:**
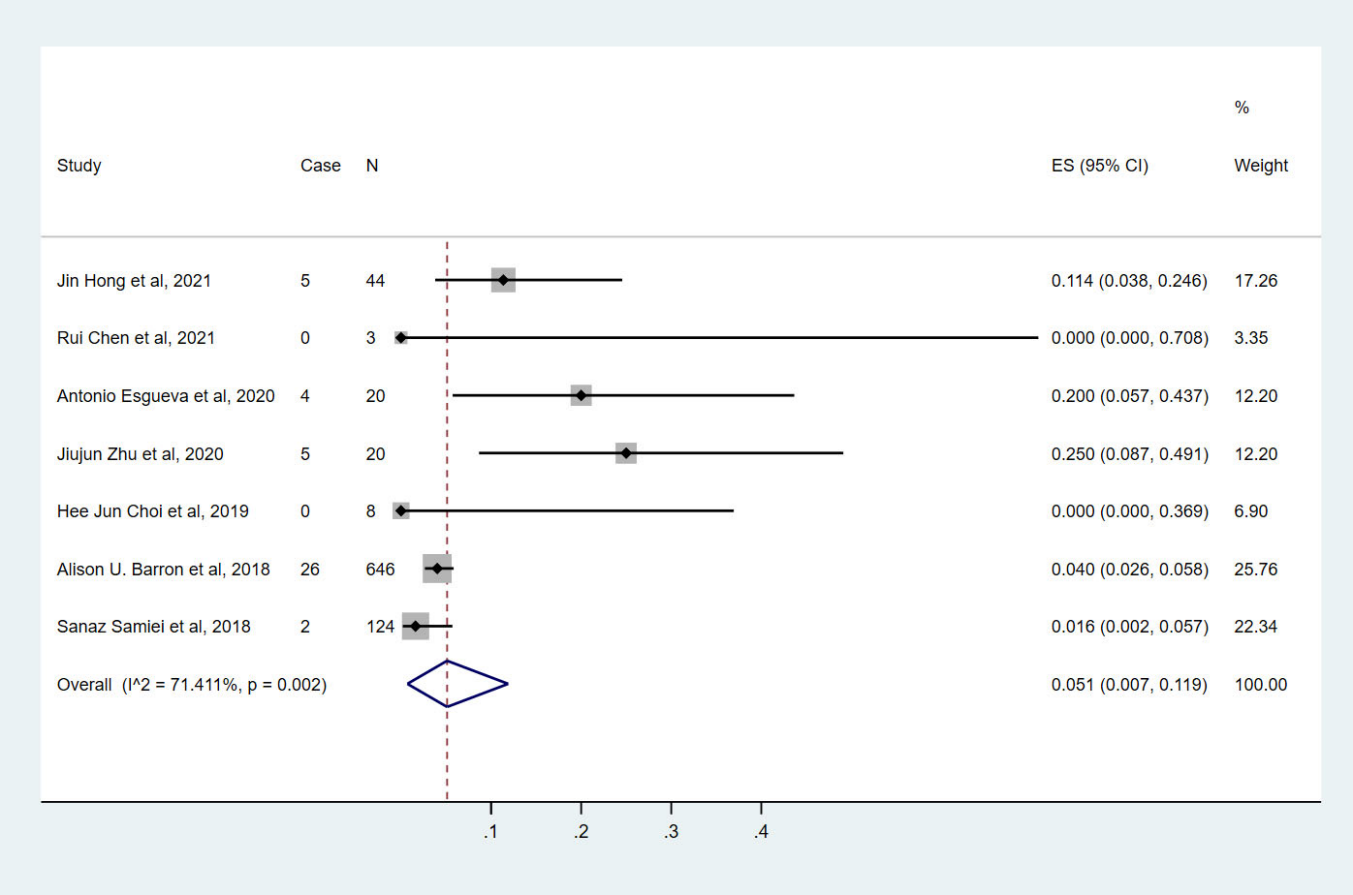
The overall pooled ypN+ rate of cN0 patients who achieved bpCR in HR+/HER2- breast cancer. HR, hormone receptor; HER2, human epidermal growth factor receptor 2. ES, effect size. CI, confidence interval. Effect size was used to estimate the ypN+ rate of each study. Confidence intervals determine the consistency and reliability of the mean estimated effect size. Diamonds indicate effect size.

**Figure 3:**
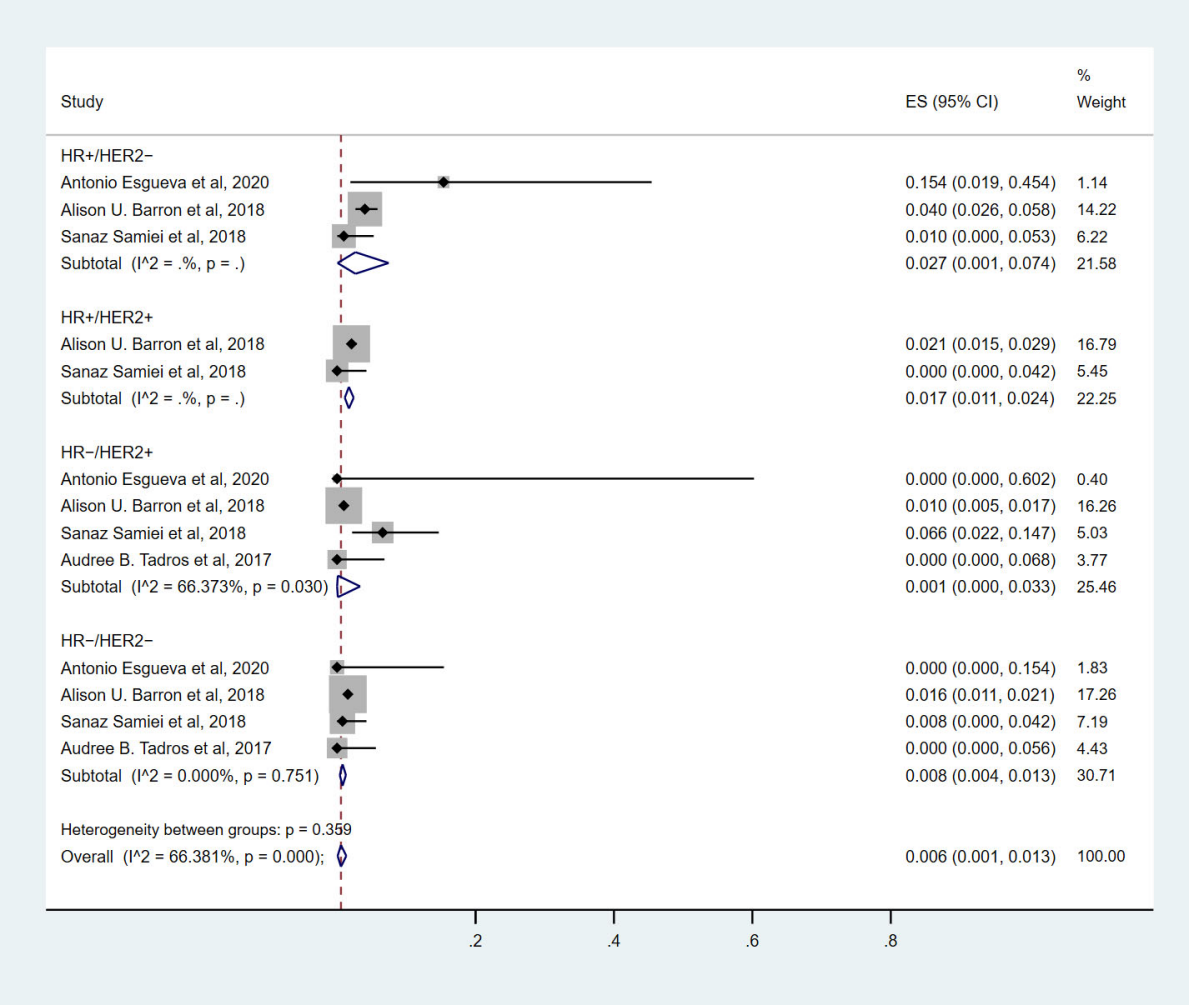
The overall pooled ypN+ rate of cN0 patients who achieved bpCR for different breast cancer subtypes with T1-T2 tumors. HR, hormone receptor; HER2, human epidermal growth factor receptor 2. ES, effect size. CI, confidence interval. Effect size was used to estimate the ypN+ rate of each study. Confidence intervals determine the consistency and reliability of the mean estimated effect size. Diamonds indicate effect size.

Seven studies (9, 10, 14–18) involving 865 cN0 patients with bpCR who had HR+/HER2- breast cancer were published (**Figure 2**). The overall pooled ypN+ rate was 5.1% (95% CI, 0.7%-11.9%) (42/865 cases). With an I^2^ value of 71.41% between the trials, there was significant heterogeneity (P=0.002). According to three studies (9, 10, 15) (**Figure 3**), 762 cT1-T2N0 patients who achieved bpCR had a pooled ypN+ rate of 2.7% (95% CI, 0.1%-7.4%) (29 cases).

### HR+/HER2+ breast cancer

**Figure 4:**
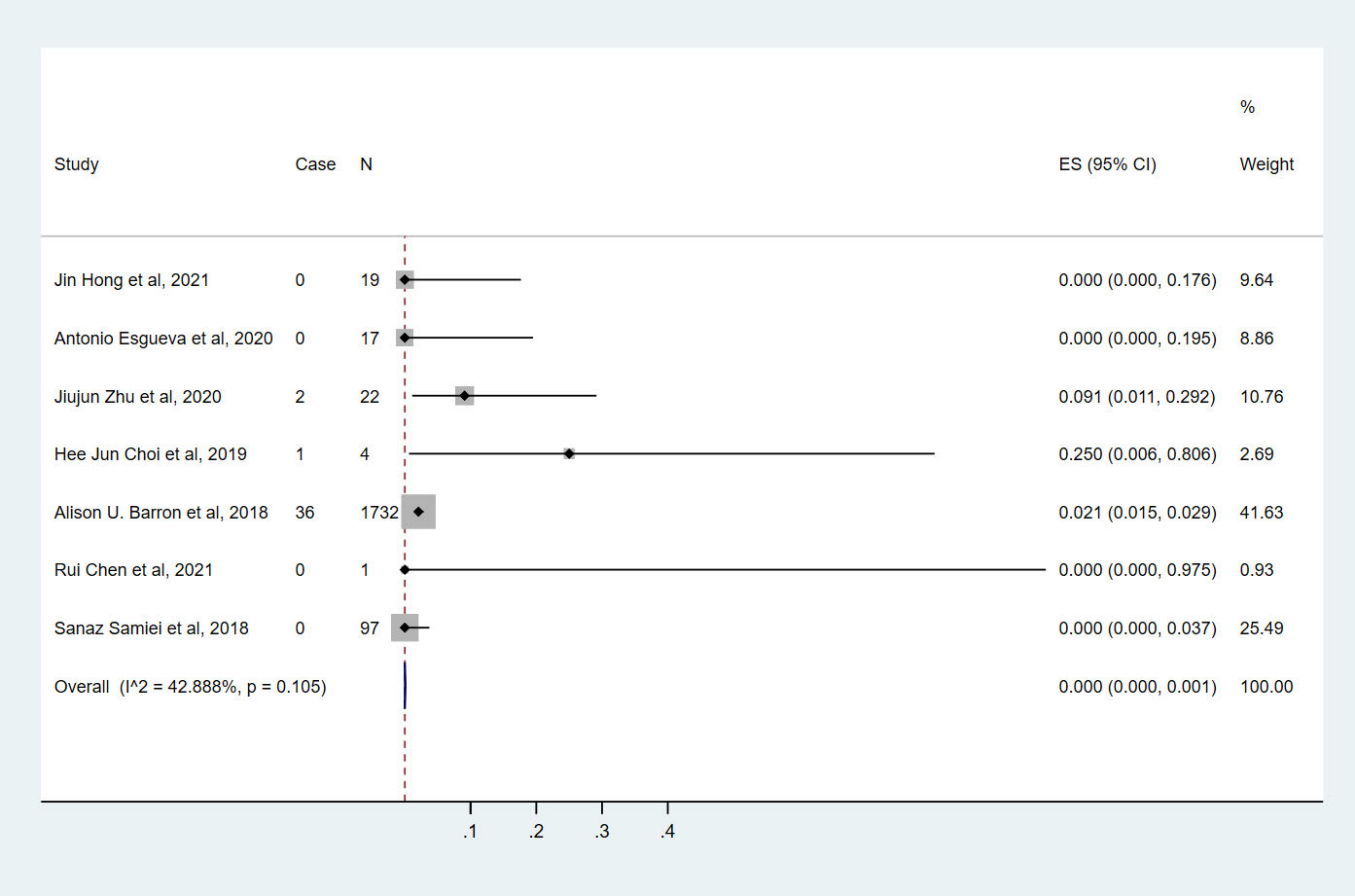
The overall pooled ypN+ rate of HR+/HER2+ breast cancer cN0 patients who achieved bpCR. HR, hormone receptor; HER2, human epidermal growth factor receptor 2. ES, effect size. CI, confidence interval. Effect size was used to estimate the ypN+ rate of each study. Confidence intervals determine the consistency and reliability of the mean estimated effect size. Diamonds indicate effect size.

Seven studies (9, 10, 14–18) involving 1892 cN0 patients with bpCR who had HR+/HER2+ breast cancer were published (**Figure 4**). The overall pooled ypN+ rate was 0% (95% CI, 0%-0.1%) (39/1892 cases). With an I^2^ value of 42.89% between the trials, there was no significant difference in heterogeneity (P=0.105). According to two studies (9, 10) (**Figure 3**), 1817 cT_1_-T_2_N0 patients who achieved bpCR had a pooled ypN+ rate of 1.7% (95% CI, 1.1%-2.4%) (36/1817 cases).

### HR-/HER2+ breast cancer

**Figure 5:**
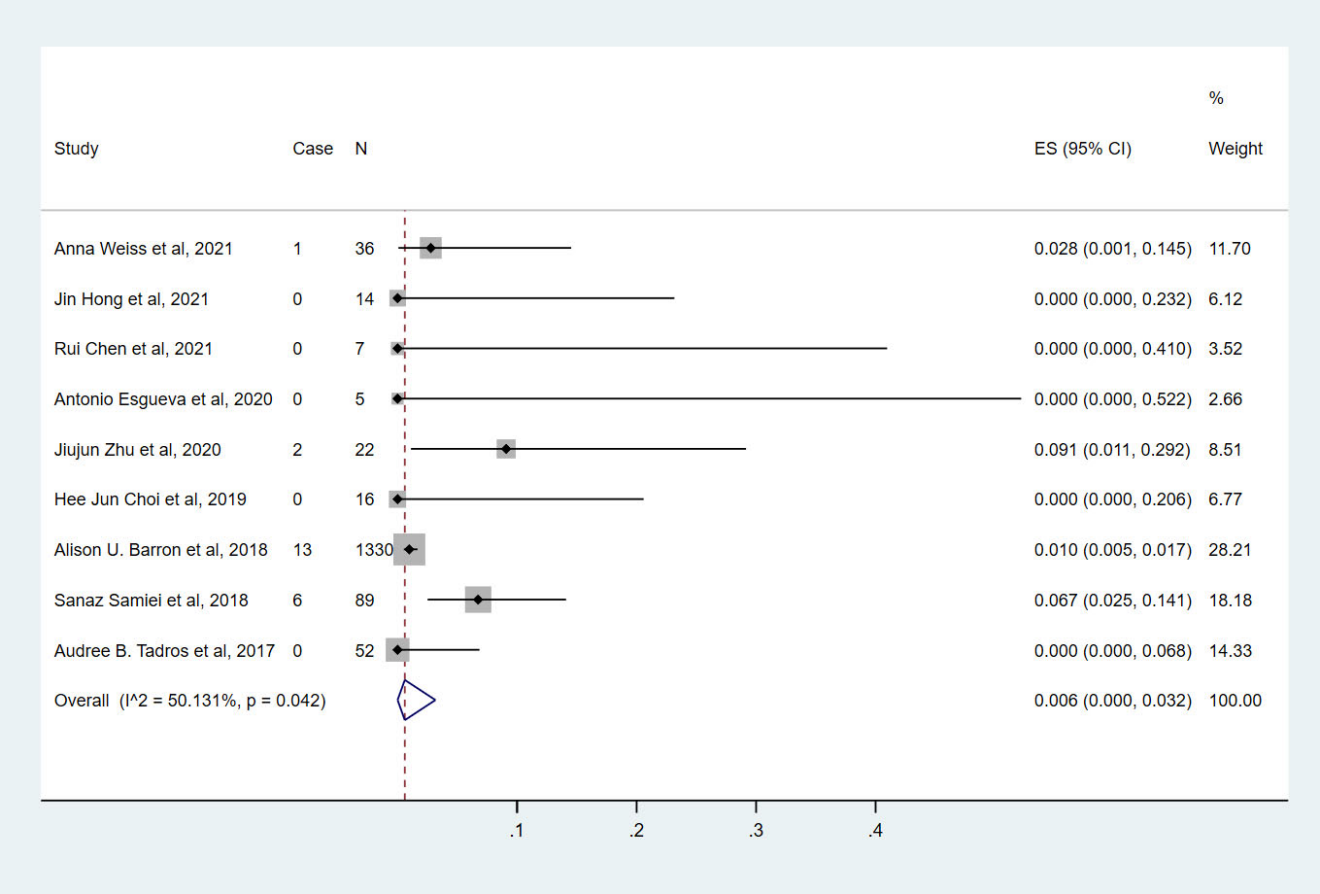
The overall pooled ypN+ rate of cN0 patients who achieved bpCR in HR-/HER2+ breast cancer. HR, hormone receptor; HER2, human epidermal growth factor receptor 2. ES, effect size. CI, confidence interval. Effect size was used to estimate the ypN+ rate of each study. Confidence intervals determine the consistency and reliability of the mean estimated effect size. Diamonds indicate effect size.

Nine studies (7, 9, 10, 13–18) involving 1571 cN0 patients with bpCR who had HR-/HER2+ breast cancer were published (**Figure 5**). The overall pooled ypN+ rate was 0.6% (95% CI, 0%-3.2%) (22/1571 cases). With an I^2^ value of 50.13% between the trials, there was significant heterogeneity (P=0.042). According to four studies (7, 9, 10, 15) (**Figure 3**), 1428 cT_1_-T_2_N0 patients who achieved bpCR had a pooled ypN+ rate of 0.1% (95% CI, 0%-3.3%) (16/1462 cases). With an I^2^ index of 66.37%, significant heterogeneity was observed (P=0.030).

### HR-/HER2- breast cancer

**Figure 6:**
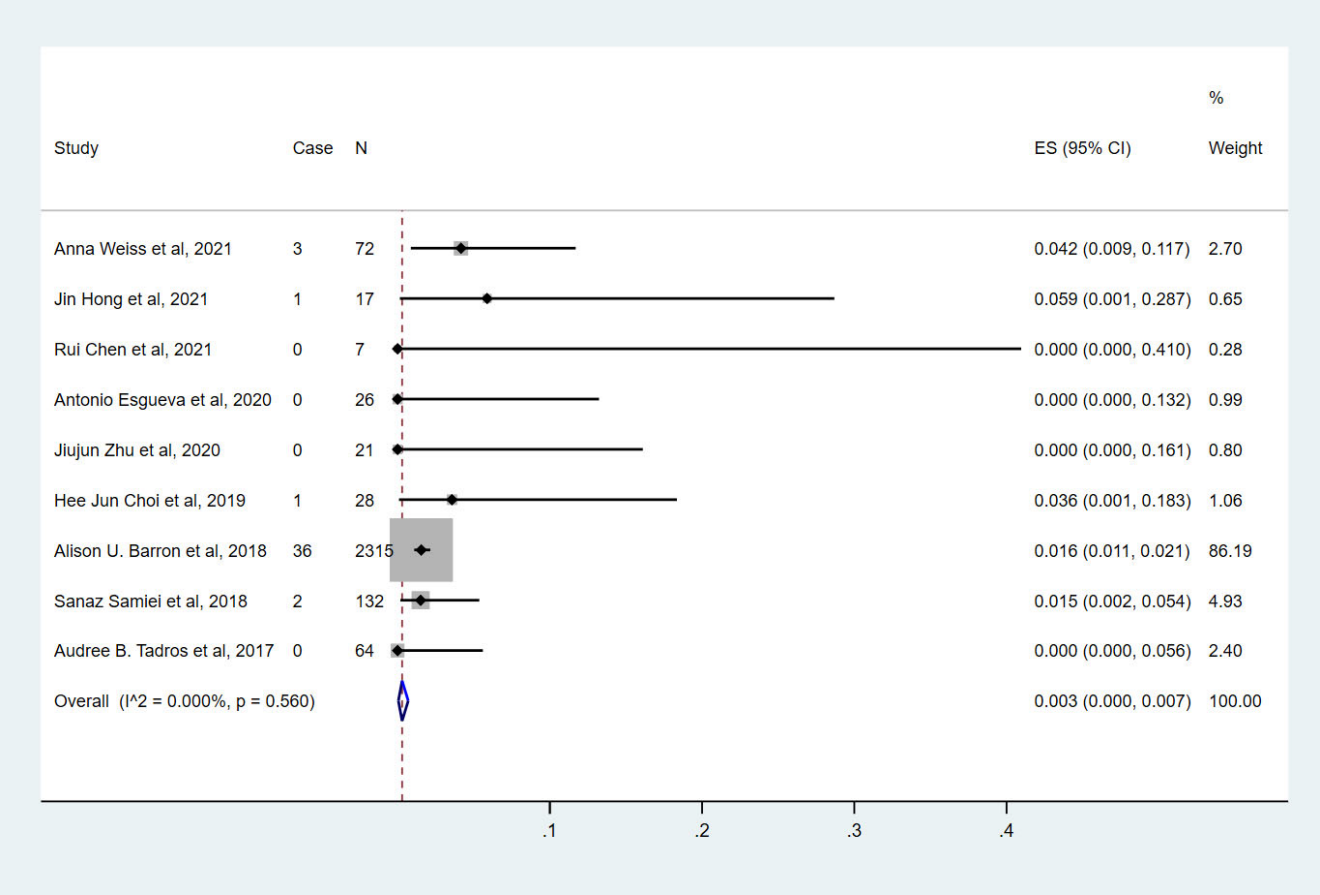
The overall pooled ypN+ rate of cN0 patients who achieved bpCR in HR-/HER2- breast cancer. HR, hormone receptor; HER2, human epidermal growth factor receptor 2. ES, effect size. CI, confidence interval. Effect size was used to estimate the ypN+ rate of each study. Confidence intervals determine the consistency and reliability of the mean estimated effect size. Diamonds indicate effect size.

Nine studies (7, 9, 10, 13–18) involving 2682 cN0 patients with bpCR who had HR-/HER2- breast cancer were published (**Figure 6**). The overall pooled ypN+ rate was 0.3% (95% CI, 0%-0.7%) (43/2682 cases). With an I^2^ value of 0% between the trials, no statistically significant heterogeneity was observed (P=0.560). According to four studies (7, 9, 10, 15) (**Figure 3**), 2530 cT_1_-T_2_N0 patients who achieved bpCR had a pooled ypN+ rate of 0.8% (95% CI, 0.4%-1.3%) (37/2530 cases). With an I^2^ index of 0%, there was no significant heterogeneity (P=0.751).

## Discussion

To our knowledge, this is the first systematic review and meta-analysis to investigate the ypN+ rate of cN0 patients with bpCR after NST in different breast cancer subtypes. The overall pooled analysis of 9609 cN0 patients who achieved bpCR showed that the ypN+ rate was only 0.2%, and the ypN+ rate was the lowest for the HR+/HER2+ (0%) subtype, followed by the HR-/HER2- (0.3%), HR-/HER2+ (0.6%), and HR+/HER2- (5.1%) subtypes.

At present, cN0 is determined by clinical physical examination, imaging and fine needle aspiration biopsy or core needle biopsy, an assessment that is accompanied by a certain FNR before NST. Although approximately 30% of patients have axillary lymph node metastases prior to NST, only 2-6% of these patients who achieve bpCR remain SLNB positive after NST, and the rate is even lower in HER2-positive and TNBC patients (7, 9, 10, 13–19). A retrospective study (10) from the National Cancer Database (NCDB) revealed that in patients with cT1/cT2 N0 HER2-positive cancer or TNBC who attained bpCR, the nodal positivity rate was less than 2%, which supports the idea of forgoing axillary surgery in this population of patients. Another retrospective study (9) that included patients with cT1-3N0-1 breast cancer from the Netherlands Cancer Registry also revealed that the rates of ypN+ for the HR+HER2+ (1.6%), HR-/HER2- (1.5%), and HR-/HER2+ (0%) subtypes were incredibly low. Furthermore, Tadros et al. (7) demonstrated that bpCR has a significant correlation with axillary nodal status following NST. The application of SLNB omission in HR+/HER2- was constrained by the significantly lower overall rate of bpCR. Moreover, NST is also utilized less frequently in cN0 individuals with HR+/HER2- illness. Therefore, the analysis or decision of whether it is safe for such patients to be exempt from SLNB must be made with extra caution. In summary, individuals with bpCR had a nodal positivity rate of less than 10%, which is in favor of exempting axillary surgery in this population of patients. For HR-/HER2-, HER2+ breast cancer, ypN+ rates were even lower (less than 2%), and patients with these two subtypes can be relieved of axillary surgery. In light of the aforementioned findings, future clinical trials should investigate whether axillary surgery can be safely omitted in precisely chosen patients.

However, in clinical practice, there will be some problems. First, for breast cancer patients with non-bpCR, the ypN+ rate of each subtype was more than 10%, except for some studies about HR-/HER2+ and HR-/HER2- (**Supplementary Table 2**), and there is no evidence to suggest that it is safe to exempt axillary surgery for these patients. Residual disease can provide guidance for patients in their decisions about adjuvant systemic therapy. Studies have confirmed that HER2+ patients with non-bpCR after neoadjuvant chemotherapy can subsequently be treated with adjuvant trastuzumab emtansine (T-DM1), while HR-/HER2- patients can be treated with capecitabine, which can improve event-free survival or disease-free survival (20, 21). Therefore, for non-bpCR patients, SLNB is still required to confirm axillary lymph node metastases; otherwise, false-negative pathological complete response assessment would result in inappropriate de-escalation of axillary lymph node metastasis and inappropriate adjuvant therapy, which may bring a higher risk of recurrence. Perhaps the findings of ongoing clinical trials (22, 23) comparing complete ALND to axillary radiotherapy in patients with positive SLNB (ypN+) following NST will provide a solution for these patients.

Second, as bpCR becomes increasingly common due to improved systemic therapy in NST, identifying bpCR prior to surgery is a critical challenge when developing surgical intervention-free treatment alternatives for individuals who have attained bpCR. Radiological CR (rCR) was considered to be used to predict bpCR and thus dispense with breast surgery; however, modern imaging techniques, including ultrasound, MRI, and F-FDG PET-CT scan, are insufficiently precise to differentiate bpCR (24–26). Henry et al. demonstrated that the vacuum-assisted breast biopsy (VABB) technique is promising for eliminating breast surgery in specific breast cancer patients after neoadjuvant chemotherapy (27). Several trials (such as MICRA) also demonstrated that VABB is not accurate enough to identify bpCR in patients with a good response on MRI after NST (28–31). Therefore, for cN0 patients who have undergone NST, breast surgery can be performed first, and the next course of axillary treatment will depend on whether the bpCR is attained. If bpCR is not achieved, axillary surgery is required as a second procedure, which is in concordance with EUBREAST-01 protocol (32). EUBREAST-01 is an ongoing international, prospective, non-randomized, single-arm surgical study. Its goal is to demonstrate the cancer-related safety of not performing axillary SLNB after achieving bpCR in response to NST for TNBC and HER2-positive patients with cN0 status (32). In this trial, axillary surgery is not carried out simultaneously. This approach offers two benefits: firstly, it shortens the duration of the operation, and secondly, it reduces the risk of lymphedema and other complications for patients who don’t undergo axillary surgery.

## Limitations

The greatest limitation in this meta-analysis is the presence of heterogeneity, the main possible causes of which are as follows. First, none of the included studies was a prospective randomized clinical trial; thus, the distribution of patients and the limited availability of details of axillary surgery might have biased our results regarding regional control. Second, information on systemic therapy was not included in our analysis because the specific chemotherapy regimen and number of patients for each subtype could not be extracted from the original literature. For example, this study included a large proportion of HER2+ patients. Targeted therapy in this population is known to increase bpCR and apCR rates, but it is not possible to obtain the proportion of targeted drug use in this population. Third, patients with cN0 confirmed with pathological biopsy before NST as well as others without such confirmation were included in the present study, but we did not have enough information to distinguish between them.

## Conclusions

In summary, the results of this meta-analysis indicated that cN0 patients who achieved bpCR may be exempt from axillary surgery in HR+/HER2+ and HR-/HER2- subtypes because of the extremely low probability of ypN+. However, it remains unclear whether the presence of axillary lymphatic disease in this specific population affects long-term survival and recurrence. Therefore, the safety of exemption from axillary surgery needs to be further confirmed by prospective studies.

## Data availability statement

The raw data supporting the conclusions of this article will be made available by the authors, without undue reservation.

## Author contributions

Concept and design: ZF, HZ and JH. The acquisition, analysis, and/or interpretation of data: LM, HC, PX and YL. Draft of the manuscript: LM, HC, PG and HZ. Supervision: ZF, HZ and JH. All other authors contributed by providing revisions to the manuscript, giving approval of the final version of the manuscript, and accepting responsibility for all aspects of the manuscript.
