# Supplementary material for: The nodal positivity rate in breast pCR patients with initially, clinically node-negative breast cancer after neoadjuvant systemic therapy: A systematic review and meta-analysis
Source: Front Oncol. 2023 Mar 29;13:1167912. doi: 10.3389/fonc.2023.1167912 (PMC10090490; doi:10.3389/fonc.2023.1167912)
Supplement: Supplementary file 1 [file DataSheet_1.docx]

**Supplementary Materials:**

**Methods**

1. **Literature search Strategy- Pubmed**

((((("Breast Neoplasms"[Mesh]) OR ((((Breast[Mesh] OR breast*[Title/Abstract] OR mamma*[Title/Abstract]))) AND (("Neoplasms"[Mesh] OR neoplas*[Title/Abstract] OR tumor[Title/Abstract] OR tumors[Title/Abstract] OR tumour[Title/Abstract] OR tumours[Title/Abstract] OR cancer*[Title/Abstract] OR malign*[Title/Abstract] OR carcinom*[Title/Abstract]))))) AND (((("Axilla"[Mesh] OR axilla*[Title/Abstract]))) OR (("Lymphatic System"[Mesh]) OR ((((lymph[Title/Abstract] OR lymphatic[Title/Abstract]))) AND ((node*[Title/Abstract] OR noda*[Title/Abstract])))))) AND (("Neoadjuvant Therapy"[Mesh]) OR ((((neoadjuvant[Title/Abstract] OR neo adjuvant[Title/Abstract] OR preoperative*[Title/Abstract] OR pre operative*[Title/Abstract]))) AND ((chemotherap*[Title/Abstract] OR chemo therap*[Title/Abstract] OR systemic[Title/Abstract] OR targeted[Title/Abstract]))))) AND ((pathologic*[Title/Abstract] OR response[Title/Abstract]))

1. **Literature search Strategy- Embased**

52. #17 AND #31 AND #47 AND #50 AND [article]/lim 1,956

51. #17 AND #31 AND #47 AND #50 4,797

50. #48 OR #49 3,726,003

49. response:ti,ab,kw 3,069,144

48. pathologic*:ti,ab,kw 746,400

47. #38 AND #46 116,298

46. #39 OR #40 OR #41 OR #42 OR #43 OR #44 OR #45 2,303,624

45. targeted:ti,ab,kw 583,740

44. systemic:ti,ab,kw 784,848

43. 'chemo therap*':ti,ab,kw 2,248

42. chemotherap*:ti,ab,kw 748,257

41. 'molecularly targeted therapy'/exp 50,400

40. 'systemic therapy'/exp 45,276

39. 'chemotherapy'/exp 766,888

38. #32 OR #33 OR #34 OR #35 OR #36 OR #37 596,852

37. 'pre operative*':ti,ab,kw 71,843

36. 'preoperative*':ti,ab,kw 514,427

35. 'neo adjuvant*':ti,ab,kw 8,411

34. neoadjuvant*:ti,ab,kw 82,302

33. 'preoperative treatment'/exp 20,149

32. 'neoadjuvant therapy'/exp 39,287

31. #18 OR #21 OR #30 1,665,375

30. #22 OR #29 1,628,289

29. #25 AND #28 345,503

28. #26 OR #27 498,146

27. noda*:ti,ab,kw 82,898

26. node*:ti,ab,kw 453,120

25. #23 OR #24 409,695

24. lymphatic:ti,ab,kw 76,293

23. lymph:ti,ab,kw 360,174

22. 'lymphatic system'/exp 1,460,618

21. #19 OR #20 67,561

20. axilla*:ti,ab,kw 64,848

19. 'axilla'/exp 12,332

18. 'axillary lymph node'/exp 20,723

17. #1 OR #16 791,043

16. #5 AND #15 678,051

15. #6 OR #7 OR #8 OR #9 OR #10 OR #11 OR #12 OR #13OR #14 6,055,185

14. carcinom*:ti,ab,kw 1,010,811

13. malign*:ti,ab,kw 942,264

12. cancer*:ti,ab,kw 3,009,690

11. tumours:ti,ab,kw 189,406

10. tumour:ti,ab,kw 330,169

9. tumors:ti,ab,kw 938,307

8. tumor:ti,ab,kw 1,848,644

7. neoplas*:ti,ab,kw 523,166

6. 'malignant neoplasm'/exp 4,240,561

5. #2 OR #3 OR #4 1,193,761

4. mamma*:ti,ab,kw 499,912

3. breast*:ti,ab,kw 723,608

2. 'breast'/exp 126,981

1. 'breast tumor'/exp 631,312

**Supplementary Table 1. Newcastle-Ottawa Scale for assessing the quality of studies in meta-analysis**

| **Study** | **Selection** | | | | **Comparability** | **Exposure** | | | **Score** |
| --- | --- | --- | --- | --- | --- | --- | --- | --- | --- |
|  | **Is the case definition adequate?** | **Representativeness of the cases** | **Selection of controls** | **Definition of controls** | **Comparability of cases and controls on the basis of the design or analysis** | **Ascertainment of exposure** | **Same method of ascertainment for cases and controls** | **Non-Response rate** |  |
| Weiss et al.^13^ | ***** | ***** | **-** | **-** | ****** | ***** | ***** | ***** | **7** |
| Hong et al.^14^ | ***** | ***** | **-** | **-** | ***** | ***** | ***** | ***** | **6** |
| Esgueva et al.^15^ | ***** | ***** | **-** | **-** | ****** | ***** | ***** | ***** | **7** |
| Choi et al.^18^ | ***** | ***** | **-** | **-** | ***** | ***** | ***** | ***** | **6** |
| Zhu et al.^17^ | ***** | ***** | **-** | **-** | ***** | ***** | ***** | ***** | **6** |
| Barron et al.^10^ | ***** | ***** | **-** | **-** | ***** | ***** | ***** | ***** | **6** |
| Chen et al.^16^ | ***** | ***** | **-** | **-** | ***** | ***** | ***** | ***** | **6** |
| Samiei et al.^9^ | ***** | ***** | **-** | **-** | ***** | ***** | ***** | ***** | **6** |
| Tadros et al.^7^ | ***** | ***** | **-** | **-** | ****** | ***** | ***** | ***** | **7** |

Note: A study can be awarded a maximum of one “*” for each numbered item within the Selection and Exposure categories. A maximum of two stars can be given for Comparability.

**Supplementary Table 2. Summary of previous studies of ypN+ rate after NST with non-bpCR.**

| **Studies** | **No. of participants** | **HR+/HER2+** | **HR+/HER2−** | **HR-/HER2+** | **HR-/HER2-** |
| --- | --- | --- | --- | --- | --- |
| Weiss et al.^13^ | 133 | - | - | 22.4% | 14.3% |
| Hong et al.^14^ | 363 | 26.9% | 41% | 17% | 29.3% |
| Esgueva et al.^15^ | 154 | 36.7% | 26.7% | 13.3% | 9.8% |
| Choi et al.^18^ | 114 | 31.8% | 41.3% | 8.3% | 14.1% |
| Zhu et al.^17^ | 316 | 34% | 54.6% | 27.1% | 34.5% |
| Barron et al.^10^ | 9482 | 18.6% | 33.1% | 11.3% | 12.6% |
| Chen et al.^16^ | 1232 | 15% | 35.3% | 9.7% | 12.7% |
| Samiei et al.^9^ | 174 | - | - | 1.3% | 9.6% |
